# Concentrated Food in the Treatment of Pulmonary Consumption1Abstract of a lecture delivered at the Philadelphia Polyclinic.

**Published:** 1891-09

**Authors:** Thomas J. Mats

**Affiliations:** Professor of Diseases of the Chest in the Philadelphia Polyclinic; and Visiting Physician to the Rush Hospital for Consumption, of Philadelphia


### CONCENTRATED FOOD IN THE TREATMENT OF PULMONARY CONSUMPTION.[1]

By THOMAS J. MAYS, M. D.,

Professor of Diseases of the Chest in the Philadelphia Polyclinic; and Visiting Physician to the Rush Hospital for Consumption, of Philadelphia.

Calling attention to the importance of nourishing diet in the treatment of pulmonary consumption, is so trite that it barely deserves repetition; yet, old as it is, it is no less true today than it ever was. Indeed, it may be laid down as a fundamental proposition, that the cases of consumption which cannot be reached through the instrumentality of food have certainly slim prospects of recovery. It is also no less true on the other hand that if your patient can be made to partake of, digest, and assimilate a sufficient amount of food, it matters little in what condition his lungs may be, he will, with ordinary good management, make a good recovery in the great majority of instances. Failure to get well under these circumstances is the exception. To make your patient eat, then, is the great problem to solve in the cure of this disease, yet everyone realizes the enor-

1. Abstract of a lectnre delivered at the Philadelphia Polyclinic.

mous difficulties which are constantly placing themselves in our way. Very little can be done to attain this end by only addressing medicines to the stomach. You are required to rise higher than this, and to take a general survey of the whole condition of your patient. In other words, it is absolutely indispensable that you should regulate his exercise, his rest, his sleep, and his eating ; in fact, you must have a systematic supervision of all he does during the whole twenty-four hours. I arrived at the conclusion, long ago, that a consumptive patient who is fatigued cannot eat. So, his appetite will greatly depend on how much or how little exercise you prescribe for him. If much exercise tires, then less must be taken, and if little exercise tires, then absolute rest must be insisted on. Many of these poor people exercise themselves to death. Digestion, like exercise, requires a certain degree of bodily strength. The strength which is expended in performing exercise deducts so much from the sum total of the bodily forces, and in most cases leaves too small a residuum to carry on the processes of digestion, absorption and assimilation, and is the principal cause of the persistent anorexia. I am well aware of the prevalent impression that exercise is one of the essential promoters of a good appetite, but all you need to do is to ask your patient to give you an opportunity to demonstrate the falsity of this belief by a prolonged dose of rest, and I dare say that a single chance will be sufficient to dispel the illusion. Rest will not only restore his appetite and save his strength, but it will reduce his fever, diminish the cough, and make him feel more comfortable in every respect.

If your patient eats, what kind of food should he have ? It is that kind which concentrates a large amount of nutritive material in a small bulk, and which requires a small amount of digestive energy on the part of the stomach and the digestive tract. Such foods exist, without question, in the freshly prepared juice of beef, oysters and clams, and they are prepared as follows: Beef, preferably the roundsteak, is cut in pieces of the size of a walnut, and is placed in a pan and held over the fire for a few minutes in order to heat the outside slightly. The whole is then dumped into a large Bartlett beef press, and this separates the juice from the fibre. About one and one-half pounds of beef will yield a teacupful of beef juice. This juice, divested of all fat, is well seasoned, and taken cold in half teacupful doses, three or four times a day. In the case of oyster and clam juice, the same process is followed in extraction, and it is likewise taken cold and seasoned. These juices contain the very essence of nourishment, require very little or no digestion, are easily absorbed and assimilated, and may be administered to the most fastidious stomachs. They are very much superior to any kind of beef tea, or extract, that can be made. Additionally, I prescribe five or six glasses of milk a day. Much may be done in feeding these patients by going about it in a systematic manner. Begin at seven o'clock in the morning with a glass of milk, and repeat the same every three hours. If a whole glass is too much, be satisfied if only half a glass is taken at first. At eight o'clock administer half a teacupful of beef juice. At first this is given three times only, but as soon as possible, four times a day. If desirable, oyster or clam juice may be substituted once during the day for the beef juice. Besides, you must persuade your patient to eat an egg, or oatmeal gruel, with cream and sugar, and bread and butter, and a cup of coffee for breakfast; beefsteak, roast beef, mutton or lamb, with vegetables, for dinner, and a lighter meal for supper. Beer, wine, champagne, whisky, or brandy may also be taken in moderate quantities throughout the day.

Much can be done to stimulate the appetite; for this purpose I often give the following: ℞. Acid phosphoric dil.; acid nitro-muriatic dil.; acid sulphuric aromatic; tinc. ferri chloridi, aa fl℥ss. M. Sig.—thirty drops in half a glass of cold sweetened water during meals. A coated tongue, which so frequently exists in these cases, is no contra-indication to the giving of iron. Additionally, two or three grains of quinine are prescribed in the forenoon and in the afternoon. The bowels must also be kept regular. If constipated, a glass of Hunyadi water, or a Lady Webster's pill in the evening, will generally suffice. Topliff's pavara pills, or Parke Davis & Co.'s cascara cordial, also serve well for this purpose. Occasionally, a blue mass pill will not be out of place. If there is a tendency to diarrhea, the above-mentioned acid preparation will often check it. In most instances of this kind, the diarrhea follows a meal, and is due more to a hyper-sensitiveness of the alimentary tract than to any other cause. To the acid mixture you may, therefore, add subnitrate of bismuth and pepsin with advantage.

---

MASTITIS.—In the Columbia Hospital for Women (*Obs. Gaz.*) a liniment composed of half an ounce of camphor dissolved in three ounces of turpentine has been found most effective in checking the secretion of milk in mastitis; it alleviates pain, lessens induration, and is more effective in reducing inflammation than any other remedy that has been tried.

